# Developing a risk score using liquid biopsy biomarkers for selecting Immunotherapy responders and stratifying disease progression risk in metastatic melanoma patients

**DOI:** 10.1186/s13046-025-03306-w

**Published:** 2025-02-05

**Authors:** Amalia Azzariti, Simona De Summa, Tommaso M. Marvulli, Ivana De Risi, Giuseppe De Palma, Roberta Di Fonte, Rossella Fasano, Simona Serratì, Sabino Strippoli, Letizia Porcelli, Michele Guida

**Affiliations:** 1 Experimental Pharmacology Laboratory, IRCCS Istituto Tumori Giovanni Paolo II, V.le O. Flacco, 65, Bari, 70124 Italy; 2 Biostatistic and Bioinformatic Laboratory, IRCCS Istituto Tumori Giovanni Paolo II, Bari, Italy; 3 Molecular Diagnostics and Pharmacogenetics Unit, IRCCS Istituto Tumori Giovanni Paolo II, Bari, Italy; 4 Rare Tumors and Melanoma Unit, IRCCS Istituto Tumori Giovanni Paolo II, Bari, Italy; 5 Biobank, IRCCS Istituto Tumori Giovanni Paolo II, Bari, Italy

**Keywords:** Metastatic melanoma, Predictor of anti-PD1 response, Anti-PD1 resistance, sPD1, sPD-L1, sLAG-3, sCTLA-4, sCD4, sCD73, And sCD74

## Abstract

**Background:**

Despite the high response rate to PD-1 blockade therapy in metastatic melanoma (MM) patients, a significant proportion of patients do not respond. Identifying biomarkers to predict patient response is crucial, ideally through non-invasive methods such as liquid biopsy.

**Methods:**

Soluble forms of PD1, PD-L1, LAG-3, CTLA-4, CD4, CD73, and CD74 were quantified using ELISA assay in plasma of a cohort of 110 MM patients, at baseline, to investigate possible correlations with clinical outcomes. A clinical risk prediction model was applied and validated in pilot studies.

**Results:**

No biomarker showed statistically significant differences between responders and non-responders. However, high number of significant correlations were observed among certain biomarkers in non-responders. Through univariate and multivariate Cox analyses, we identified sPD-L1, sCTLA-4, sCD73, and sCD74 as independent biomarkers predicting progression-free survival and overall survival. According to ROC analysis we discovered that, except for sCD73, values of sPD-L1, sCTLA-4, and sCD74 lower than the cut-off predicted lower disease progression and reduced mortality. A comprehensive risk score for predicting progression-free survival was developed by incorporating the values ​​of the two identified independent factors, sCTLA-4 and sCD74, which significantly improved the accuracy of outcome prediction. Pilot validations highlighted the potential use of the risk score in treatment-naive individuals and long responders.

**Conclusion:**

In summary, risk score based on circulating sCTLA-4 and sCD74 reflects the response to immune checkpoint inhibitor (ICI) therapy in MM patients. If confirmed, through further validation, these findings could assist in recommending therapy to patients likely to experience a long-lasting response.

**Supplementary Information:**

The online version contains supplementary material available at 10.1186/s13046-025-03306-w.

## Background

Despite the high response rate to immunotherapy with immune checkpoint inhibitors in patients with metastatic melanoma, whether BRAF wild type (wt) or mutated, there is still a significant percentage of patients who do not respond to this therapy. This represents a dual challenge: on one hand, the side effects associated with immunotherapy can compromise the patients’ quality of life; on the other hand, the high cost of these therapies burdens the National Health System (NHS) without providing tangible benefits to all treated patients.

Developing reliable methods to predict patient response to immunotherapy is crucial. A major advancement in this area can be achieved using biomarkers in human fluids, known as Liquid Biopsy. Recent technological breakthroughs have made it possible to evaluate circulating components, including tumor and immune cells, soluble factors, extracellular vesicles, tumor DNA and non-coding RNAs, all deriving from cancer cells themselves as well as from the tumor microenvironment and even in low abundance [1].

Our interest in this field began a few years ago and initially focused on the use of circulating extracellular vesicles (EVs) as factors useful for selecting patients with metastatic melanoma for immunotherapy treatment. We demonstrated that certain subpopulations of EVs are independent factors predicting the response to anti-PD1 immune checkpoint inhibitors [2, 3]. The further developing will focus on examining the role of EVs in other tumor pathologies sensitive to immunotherapy and on developing a Point-of-Care (PoC) tool for the detection of these vesicles, with the aim of making their dosage accessible in all hospitals. Nonetheless, we recognize the challenges associated with EV use as biomarkers of response, including the complexity of methods for their detection and isolation from peripheral blood.

Therefore, we have focused on the possibility of measuring other circulating biomarkers as indicators of response/resistance to anti-PD1 and of developing new tool to improve prediction of clinical outcome. After quantifying the amount of specific molecules present in peripheral blood, both immune checkpoints directly or indirectly linked to anti-PD1 therapy such as sPD1 (Soluble Programmed Cell Death Protein 1), sPD-L1 (Soluble Programmed Death-Ligand 1), sLAG-3 (Soluble Lymphocyte-Activation Gene 3), and sCTLA4 (Soluble Cytotoxic T-Lymphocyte Antigen 4) and other immune biomarkers such as sCD4 (Soluble CD4), sCD73 (Soluble CD73), and sCD74 (Soluble CD74) we interpreted their predictive values on patient outcome by developing a risk model for tumor progression.

Using ELISA kits and biostatistical approaches for mono- and multiparametric analysis, we aim to provide a straightforward approach to predict clinical outcomes using biological biomarkers.

The choice of the biomarkers is justified by their established roles in tumor immunotherapy responses. Data from literature suggested that soluble form of immune checkpoints such as sPD1, sPD-L1, sLAG-3, and sCTLA-4 levels can potentially serve as predictive biomarkers for anti-PD1 immunotherapy because they reflect the immunosuppressive tumor microenvironment and T cell dysfunction. sPD1 is the soluble form of the PD1 receptor, which binds to PD-L1 and PD-L2 ligands. Elevated levels of sPD1 have been associated with poor prognosis and resistance to anti-PD1 therapy. It competes with membrane-bound PD1 for binding to PD-L1, thus potentially limiting the efficacy of anti-PD1 therapy [4]. Like sPD1, sPD-L1 is the soluble form of the PD-L1 ligand. High levels of sPD-L1 have been correlated with tumor progression and resistance to anti-PD1 therapy. sPD-L1 can bind to PD1 and block the interaction between membrane-bound PD-L1 and PD1, thereby interfering with the mechanism of action of anti-PD1 therapy [5]. LAG-3 is a checkpoint receptor expressed on activated T cells and regulatory T cells. It negatively regulates T cell activation and proliferation. High levels of LAG-3 expression have been associated with T cell exhaustion and resistance to anti-PD1 therapy, suggesting that LAG-3 expression levels may predict response to anti-PD1 therapy [6]. The soluble form of LAG-3 (sLAG-3) has been suggested to be a co-inhibitor of immune responses by hindering monocyte differentiation and their antigen-presenting capacity, which in turn inhibits the induction of T cell proliferation [7]. CTLA-4 is another immune checkpoint receptor expressed on activated T cells. It competes with the co-stimulatory receptor CD28 for binding to CD80 and CD86 ligands on antigen-presenting cells, leading to T cell inhibition. Preclinical and clinical studies have demonstrated that tumors with high CTLA-4 expression may be more responsive to anti-PD1 therapy [8]. Some cancer cells have been found to naturally produce the soluble form of CTLA-4 (sCTLA-4), which is predicted to dampen T cell effector activity, by modulating T cell activation and altering the phenotype of intratumoral CD8^+^ T cells, and facilitate immune escape [9].

Due to their pivotal roles in modulating the immune response, promoting tumor progression, and facilitating immune evasion across various cancer types, we also focused on soluble CD4 (sCD4), soluble CD73 (sCD73) and soluble CD74 (sCD74). sCD4 is a fragment of the CD4 molecule, which is primarily known for its role in the immune system as a co-receptor that aids in the recognition of antigens presented by MHC class II molecules. sCD4 can interfere with the normal function of membrane-bound CD4^+^ T cells. It may act as a decoy receptor, binding to MHC class II molecules and preventing CD4^+^ T cells from effectively recognizing and responding to tumor antigens [10]. High levels of sCD4 can indicate immune system dysregulation associated with cancer [11]. CD73, also known as ecto-5’-nucleotidase, is an enzyme that converts extracellular AMP to adenosine, an immunosuppressive molecule [12]. sCD73 plays a significant role in the tumor microenvironment, contributing to the production of adenosine, which can inhibit T cell activation and proliferation, promoting regulatory T cell (Treg) function, and suppressing anti-tumor immune responses. Thus, sCD73 helps tumors evade immune detection and destruction. Moreover, it has been demonstrated that high levels of sCD73 are often associated with poor prognosis in various cancers because, by creating an immunosuppressive microenvironment, it facilitates tumor growth and metastasis [13]. Finally, CD74 is involved in antigen presentation by stabilizing MHC class II molecules and facilitating their transport to the cell surface [14]. sCD74 can interact with macrophage migration inhibitory factor (MIF), leading to activation of signaling pathways that promote cancer cell survival and proliferation. Elevated levels of sCD74 have been found in various cancers and are associated with tumor progression and poor prognosis, because sCD74 can contribute to an environment that supports cancer cell growth and like its membrane-bound form, sCD74 can influence immune responses. It may modulate the activity of immune cells within the tumor microenvironment, contributing to immune evasion [15].

## Methods

### Aim

We aimed to investigate the relevance of several circulating biomarkers in predicting the clinical response of MM patients treated with anti-PD1 immunotherapy.

### Study design

The study is divided into three primary analytical sections: (1) evaluating the soluble forms of PD1, PD-L1, LAG-3, CTLA-4, CD4, CD73, and CD74 as potential predictive biomarkers for the response to anti-PD1 therapy; (2) identifying independent biomarkers that can predict the response to anti-PD1 treatment; and (3) developing a risk score for progression-free survival (PFS) by integrating all independent factors derived from the multivariate analysis to provide a comprehensive assessment of the risk of tumor progression.

### Patient enrolment

The study was previously approved by the Ethics Committee of the IRCCS Istituto Tumori Giovanni Paolo II (Prot. 590/CE) and written informed consent was obtained from all the enrolled patients. Blood samples were collected from 110 MM patients treated with checkpoint inhibitors at IRCCS Istituto Tumori Giovanni Paolo II from January 2017 to December 2021 whose characteristics are reported in Table 1. Pre-therapy (basal) samples were from all MM patients: 39 responders (RES) and 71 non-responders (NRES); serial samples were obtained from 36 MM patients treated with anti-PD1 (18 NRES, 12 long RES and 6 RES > PD who initially responded to ICI and then progressed). Clinical features of the enrolled patients were collected and reported in Table 1.

**Table 1:** Main characteristics of patients at immunotherapy (*n* = 110)

Characteristics	*n* (%)
Age at metastasis, years, median [range]	58 [29–92]
Sex	
Male	60 (55)
Female	50 (45)
Basal LDH*	
< ULN	59 (56)
> ULN	46 (44)
N of metastatic sites**	
< 3	53 (49)
*≥* 3	55 (51)
Site of melanoma	301
Cutaneous	86 (78)
Mucosal	3 (3)
Ocular	3 (5)
Unknown	14 (14)
Prior therapy for metastatic disease	47 (43)
ECOG PS***	
0	57 (53)
1	42 (39)
2	9 (8)
Stage at metastatic disease	
M1a	30 (27)
M1b	19 (17)
M1c	45 (41)
M1d	16 (15)
Therapy	
Anti-PD-1	106 (96)
Anti-PD-1 plus Anti-CTLA-4	4 (4)

* 5 patients had missing value

** 2 patients had missing value

*** 2 patients had missing value

### Blood samples

Peripheral blood was collected in EDTA tubes and plasma isolated as described in our previous study [3]. Plasma samples were stored at -80 °C in the institutional Biobank before the measurement of each marker.

### Biomarkers elisa

The concentrations of sPD-1, sPD-L1, sCTLA-4, sLAG-3, sCD4, sCD73, and sCD74 were measured using enzyme-linked immunosorbent assays (ELISA). Commercial ELISA kits (details specified in Human PDCD1 / CD279 / PD-1 ELISA Kit – LSBIO; ab277712 Human PD-L1 SimpleStep ELISA^®^ Kit, ab193707 – LAG3 Human ELISA Kit, ab213761 – Human CD73 ELISA Kit – ABCAM; Human sCD152/CTLA-4 ELISA Kit – INVITROGEN; RayBio Human CD4 ELISA Kit, RayBio Human CD4 ELISA Kit – RayBiotech) were utilized, adhering strictly to the manufacturer’s protocols. Absorbance measurements were taken using the Multiskan Sky – Thermo Scientific spectrophotometer, set to a wavelength of 450 nm. The concentrations of the target proteins were derived from standard curves constructed from known concentrations of each analyte. To ensure accuracy and reproducibility, all samples, along with standards and negative controls, were analysed in duplicate.

### Statistical analysis

Statistical significance was calculated using two-tailed t-tests, Mann–Whitney U tests and two-tailed ANOVA using GraphPad Prism V.5.0 software (GraphPad Software, San Diego, California, USA). Correlation analysis was performed using the nonparametric Spearman correlation test. Data were scaled with the ‘preprocess’ function of the R package “caret” (v.6.0) using the method “range” in the interval 1–10. In detail, through “range” method data were normalized in the range 0–1 and the values were scaled in the interval 1–10, thus data are represented as Scaled Units. “A ROC analysis to identify optimal threshold able to stratify responders/not-responder patients was performed with “pROC” (v.1.18.5) R package. Univariate and multivariate Cox hazard regression and Kaplan-Meier analyses was carried out with “survival” (v.3.7) R package. The significant features of the multivariate Cox hazard regression models and their coefficients were used to calculate risk score (RS), using the general formula:

$$\:RS={\beta\:}_{1\:}X\:{biomarker}_{1\:}+{\beta\:}_{2}X\:{biomarker}_{2}+\:{\beta\:}_{3}X\:{biomarker}_{3}$$

The 5-fold cross validation of multivariate Cox model was performed with “survcomp” (v.1.54.0) and “rsample” (v.1.2.1) R packages. Optimal cutpoint for risk score were identified with the function “surv_cutpoint” of “maxstat” (v.0.7) R package. Overall response rate analysis was performed through proportion test with “chisq.test” function of “stats” (v.4.4.0) R package. Graphs were depicted through “ggplot2” R package.

## Results

### Patient cohort enrolled in the study

Seven soluble biomarkers were analyzed in the plasma of 110 MM patients, collected prior to the initiation of immunotherapy. The patients were divided into two groups: NRES, which included patients who were intrinsically resistant to immunotherapy and those whose disease stability was less than four months (PD: n. 71 and SD < 4 months: n. 3); and RES, including patients who achieved a complete response (CR: n. 13), partial response (PR: n. 18), and those with disease stability for more than four months (SD > 4 months: n. 6). The patient characteristics are summarised in Table 1. The longitudinal study included serial samples from 36 MM patients, with their characteristics detailed in the final paragraph of the Results section.

### Development and validation of a risk assessment model for disease progression in metastatic melanoma patients

The core of this study is to develop a reliable tool for assessing the risk of disease progression in metastatic melanoma patients through a comprehensive statistical analysis.

**Fig. 1:**
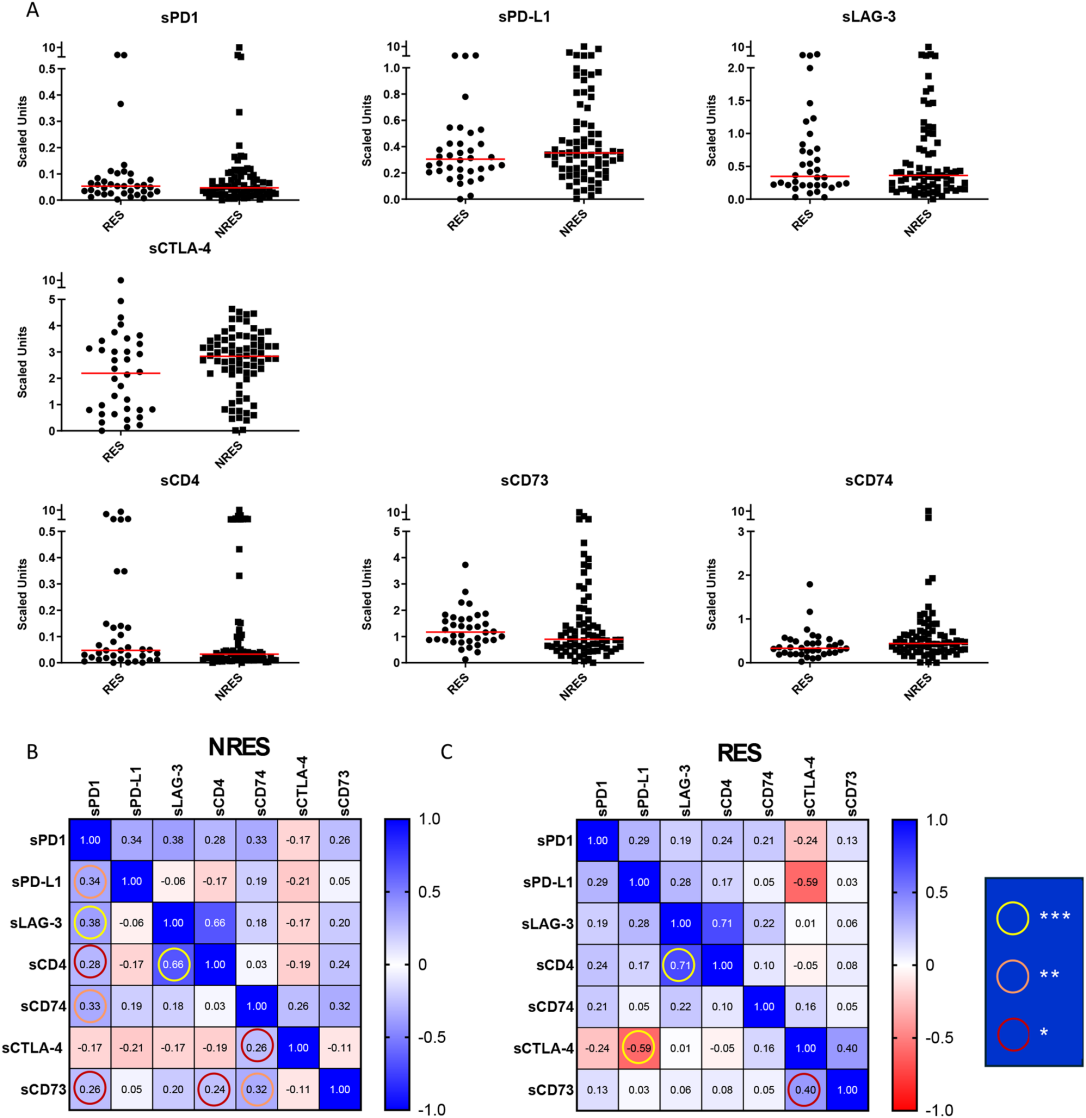
Circulating biomarkers clustered by response to therapy in MM patients and correlation analysis. (**A**) Scatter plot with median of each biomarker expressed in scaled unit from responders (*n* = 36) and non-responders (*n* = 74). (**B**) Correlogram of Spearman’s correlation coefficients between all biomarkers (*p < 0.05, **p < 0.01, *** *p* < 0.001)

For this purpose, we quantified each biomarker in the peripheral blood of RES and NRES patients using an ELISA assay and scaled the relative amounts as detailed in the Methods Section. As shown in the scatter plots, no significant differences were observed in biomarker quantities between the two patient populations, even when analyzing each response group (CR, PR, SD and PD) (Fig. 1A and S1). The results on PD1 and PD-L1 were already reported in our previous study [3]; here we investigated their involvement, together with all other biomarkers, in the response to anti-PD1 utilising univariate, the COX multivariate and the risk score analyses.

Correlation analysis revealed a higher number of significant correlations among biomarkers in NRES compared to RES, notably between sLAG-3 vs. sPD-1 and sLAG-3 vs. sCD4 (Fig. 1B and Table S1). In contrast, in RES significant correlation was found only between sPD-L1 vs. sCTLA-4 (Fig. 1C and Table S1). Highly significant correlations between sLAG-3 and sCD4 were observed in both RES and NRES groups, suggesting these factors do not play a role in determining immunotherapy response. These findings suggest that these biomarkers are more interconnected in patients in which immunotherapy is ineffective, potentially identifying individuals at higher risk of disease progression.

We then proceeded to evaluate if each marker at baseline could predict PFS and overall survival (OS) by Kaplan-Meier analysis. ROC curve analysis was performed to identify the optimal cut-off for each marker, demonstrating significant predictive relevance of marker levels for PFS and OS.

**Fig. 2:**
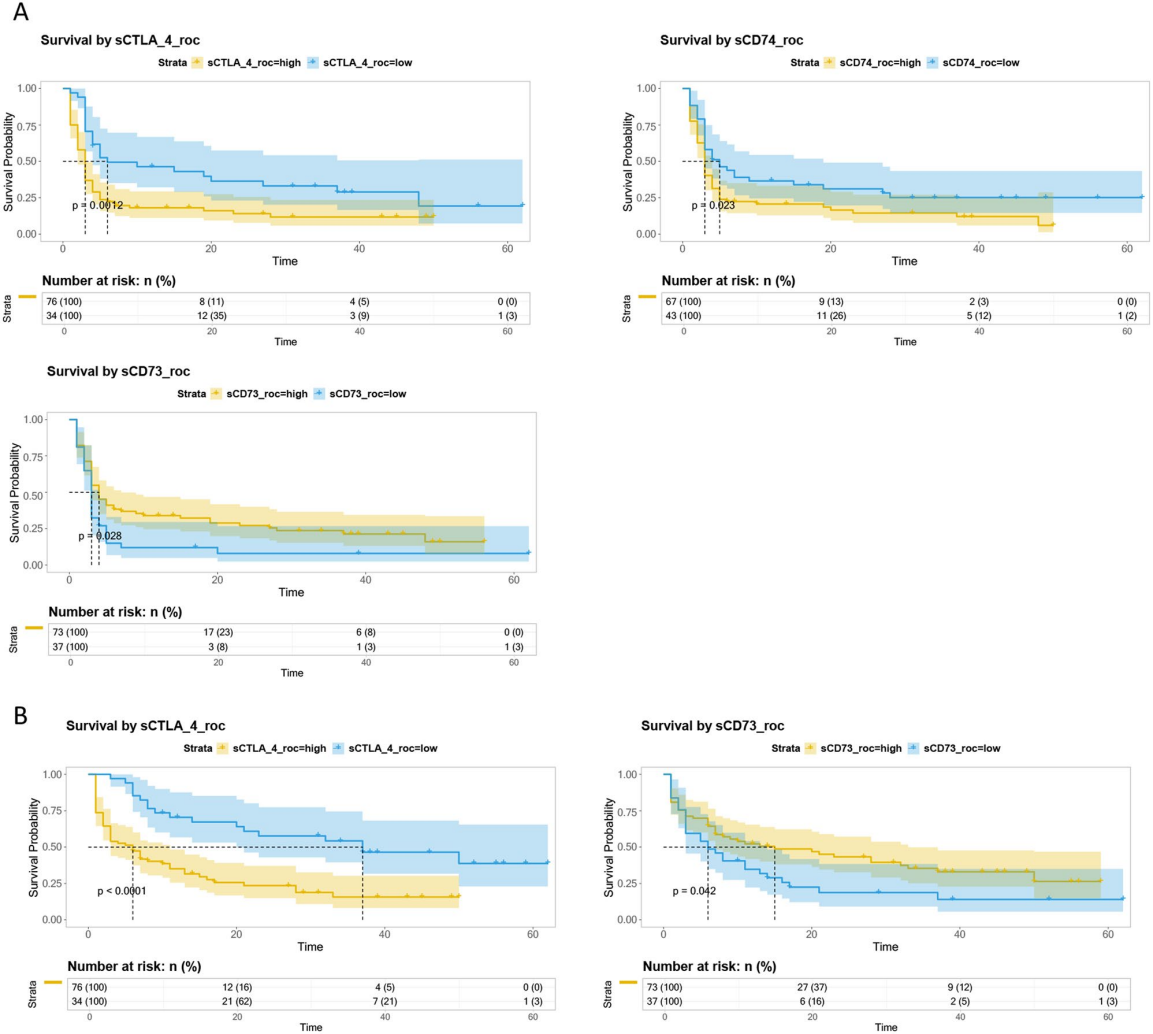
Circulating biomarkers clustered by evaluation of PFS and OS. (**A**) Statistically significant Kaplan–Meier survival curves according to each circulating biomarker clustered by ROC cut-off as respect to PFS, and (**B**) as respect to OS

**Fig. 3:**
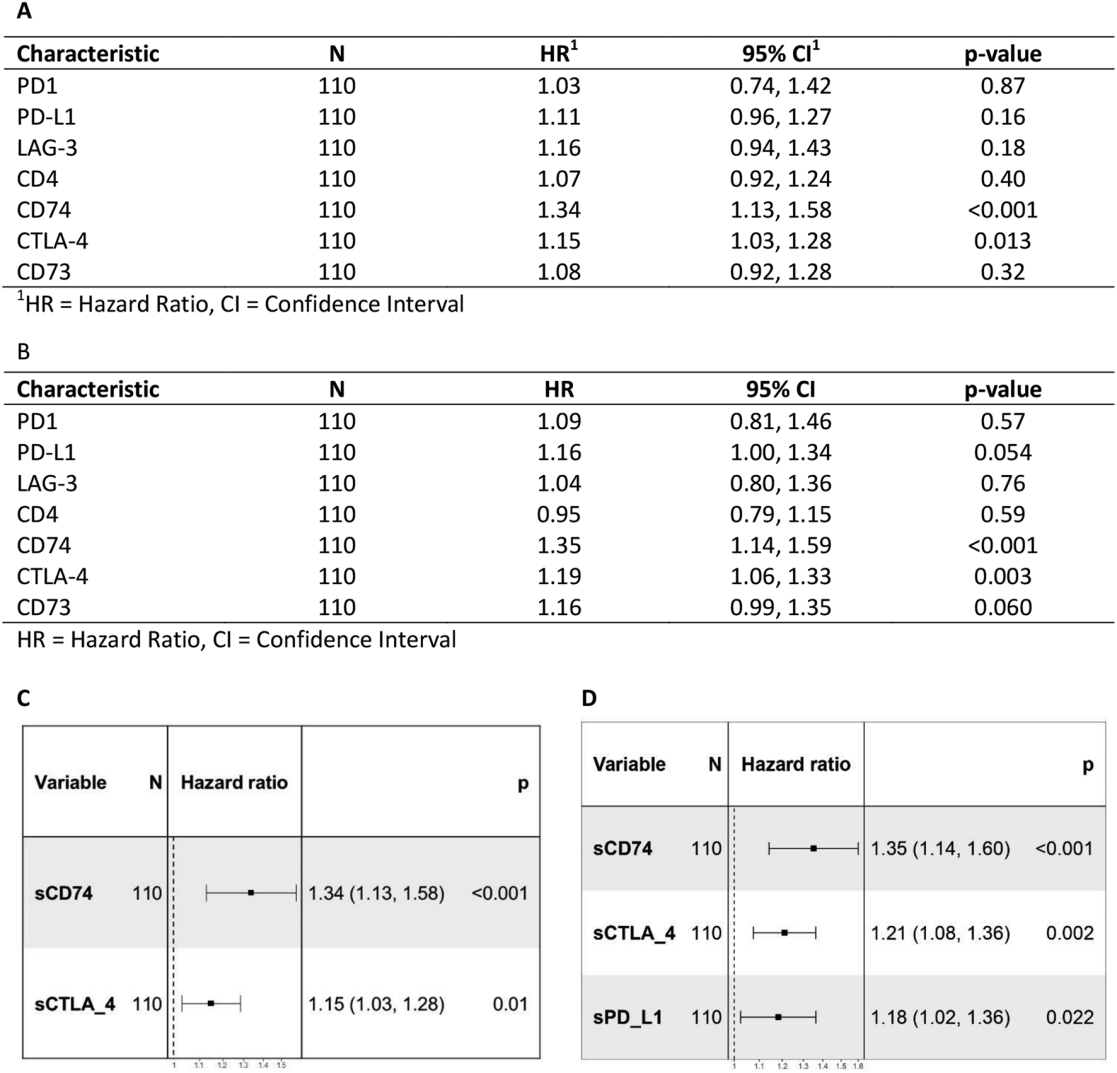
Univariate and multivariate Cox-hazard regression analysis for A/C. PFS and B/C. OS

The results highlighted the significant predictive relevance of sCTLA-4, sCD74, and sCD73 levels for PFS and sCTLA-4 and sCD73 for OS, as reported in Fig. 2, while sPD-L1, sPD1, sLAG-3 and sCD4 didn’t show it (data in Fig. S2). Lower concentrations of sCTLA-4 and sCD74 are associated with a better PFS and OS. While, considering sCD73, a better PFS is observed when sCD73 levels are higher. Next, univariate Cox regression analysis of all seven biomarkers revealed that sCTLA-4 and sCD74 are predictors for PFS, while sPD-L1, sCTLA-4, and sCD74 are predictors for OS (Fig. 3A, B).

By multivariable Cox regression analysis, the biomarkers sCD74 and sCTLA-4 were found as independent positive predictors for PFS (Fig. 3C) and for OS (Fig. 3D). In addition, sPD-L1 was another independent predictive factor for OS (Fig. 3D). Finally, through Cox Proportional Hazard model which combines the two independent factors sCD74 and sCTLA-4 for PFS into a single estimate, we found the formula for the risk score (RS) assessment of disease progression, as follows:

$$RS = {\text{ }}0.29{\text{ }}x{\text{ }}CD74{\text{ }} + {\text{ }}0,13{\text{ }}x{\text{ }}CTLA - 4$$

**Fig. 4:**
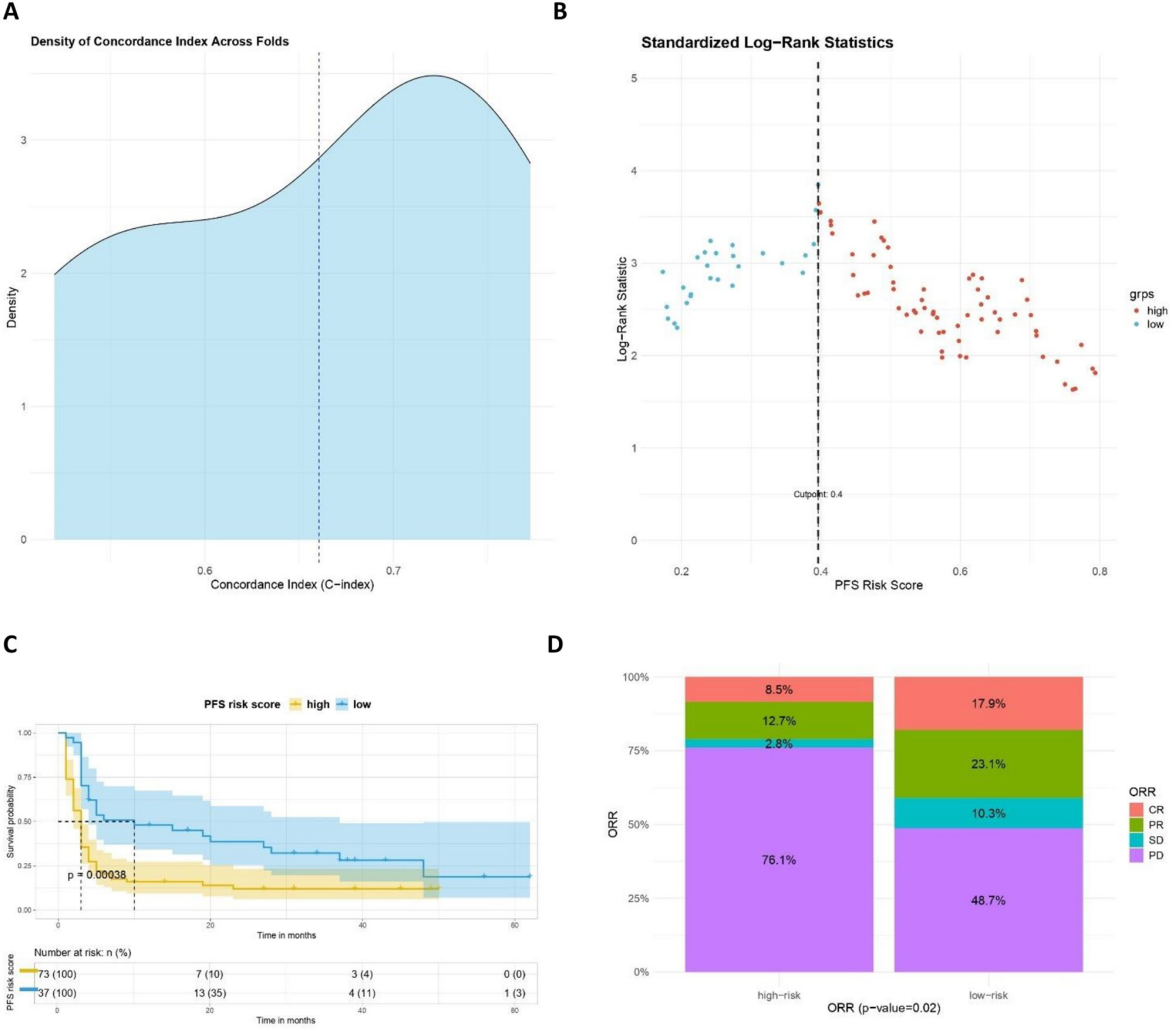
Risk score performance and survival analysis. (**A**) Density plot of the Concordance Index related to 5-fold cross validation; (**B**) Maximally selected rank statistics result to identify optimal cutpoint of the RS; (**C**) Kaplan-Meier curves comparing high/low risk patients. (**D**) Overall response rate analysis stratifying patients in high/low risk group according to previously estimated RS cutpoint

Such a model was tested with 5-fold cross validation, achieving a mean Concordance Index of 0.66, indicating a moderate predictive power, successfully ranking the predictions with a 66% accuracy (Fig. 4A).

Thus, we searched for an optimal cutpoint of the Risk Score to stratify survival curves with a method able to identify the cut-off point that maximizes the log-rank statistic. Such a cutpoint for the risk score was 0.4 as shown in Fig. 4B. According to risk score cutpoint, Kaplan-Meier curves discriminating high vs. low risk groups reached statistical significance (p value = 0.00038) and median PFS of 3 months (95% CI: 2–3) for high-risk group and of 10 months (95%CI: 4–37) for low-risk group (Fig. 4C).

Finally, overall response rate analysis was performed comparing the low vs. high risk group. As shown in Fig. 4D, there is a significant statistical difference between clinical responses, with 76.1% patients with progression disease in high-risk group vs. 48.7% in the low risk one and 8.5% CRs in high risk group vs. 17.9 CRs in low risk subset.

### Pilot validation of the risk model for tumor progression

#### In treatment-naïve MM vs. pre-treated patients

**Fig. 5:**
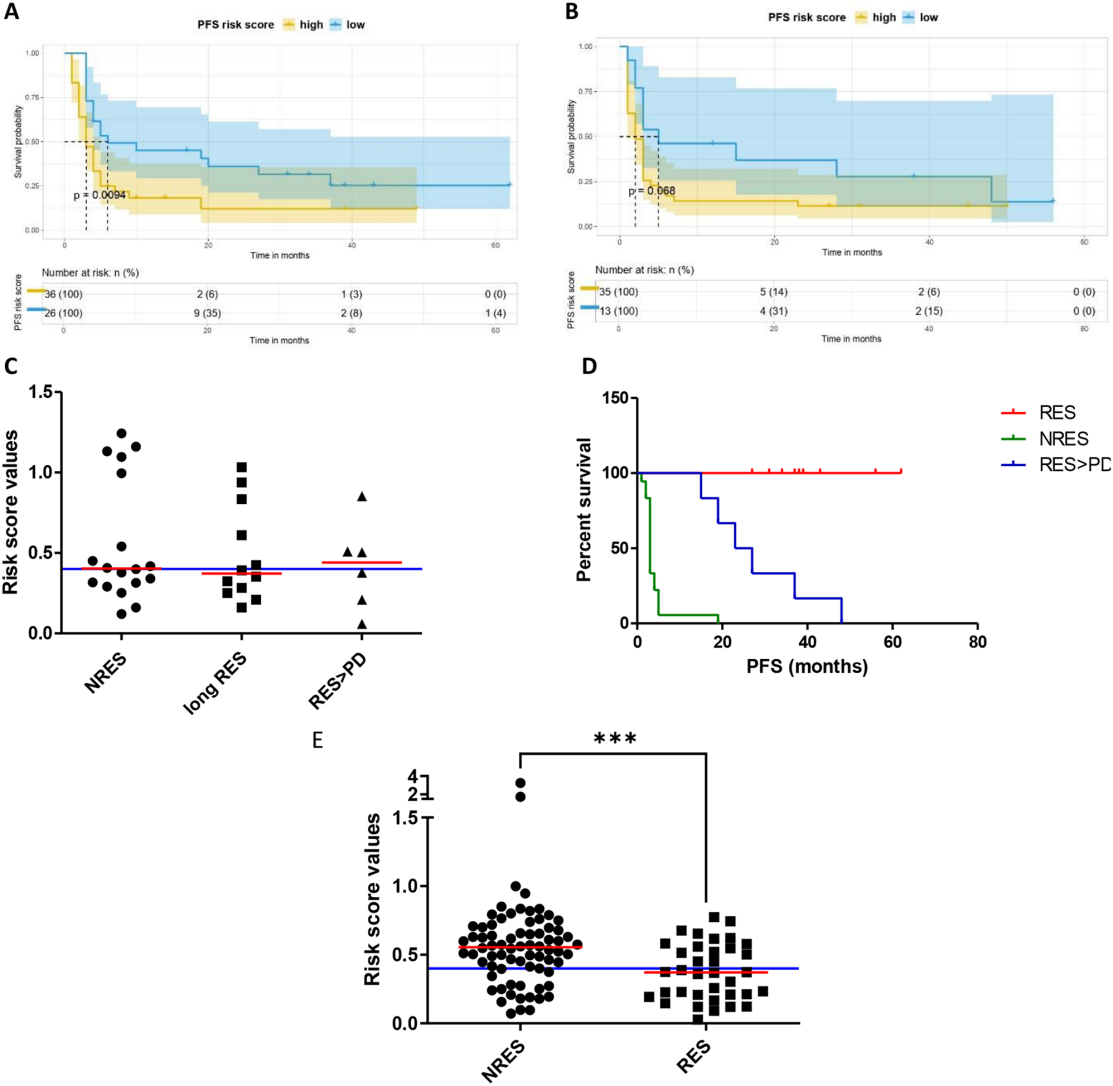
Pilot validation of the risk model for tumor progression in treatment-naïve MM vs. pre-treated patients and - In NRES vs. long RES vs. RES > PD. Statistically significant Kaplan–Meier survival curves according to RS cutpoint as respect to PFS in (**A**) treatment-naïve and (**B**) pre-treated patients with metastatic disease. (**C**) Scatter plot with median of the RS from 18 NRES, 12 long RES and 6 RES > PD. (**D**) Kaplan-Meier curves were plotted for PFS, comparing risk score values of NRES (green line), long RES (red line), and RES > PD patients (blue line). (**E**) Scatter plot with median of the RS from NRES and RES

To assess whether this RS cutpoint is effective for stratifying treatment-naïve patients with metastatic disease (26 patients with RS < 0.4 and 37 with RS > 0.4) vs. pretreated patients with metastatic disease (13 patients with RS < 0.4 and 34 with RS > 0.4), we analyzed the correlation between RS values and PFS. The results, shown in Fig. 5A, demonstrated that the model performs optimally in the treatment-naïve population, with a statistically significant difference between the two groups (RS ≥ 0.4 vs. RS < 0.4) of *p* = 0.0098 (**). Conversely, in patients who had undergone prior therapy, the KM curves for PFS between the RS ≥ 0.4 and RS < 0.4 groups showed a statistical trend (*p* = 0.068) (Fig. 5B).

#### In NRES vs. long RES vs. RES > PD

Additionally, to investigate whether the risk score model developed in this study discriminate, in addition to NRES from RES, also MM patients who progressed after a positive response to ICI. We utilized data from 36 already enrolled patients, divided into three groups: 18 NRES (patients who showed no response to anti-PD1 at the first response evaluation), 12 long RES (patients with a sustained response lasting between 4 and 18 months), and 6 RES > PD (patients who initially responded positively to the therapy but later experienced disease progression) because for each of them we had serial plasma samples in function of time.

We applied the risk score model for these three MM patient populations, utilising the baseline samples, and as expected, the NRES group had a median risk score higher than the long RES group, with the median values of 0.4031 vs. 0.3724 for PFS (Fig. 5C). The RES > PD group had a higher risk score than the RES group, 0.4411 vs. 0.3724 for PFS, categorizing these patients similarly to the NRES group (Fig. 5C). Considering the Kaplan-Meier plot of these three groups of patients, as expected, they fall into three distinct curves that are statistically different from each other (Fig. 5D). Because the analysis revealed that the distribution within the NRES group might be bimodal, to determine whether these patients can indeed be categorized into two distinct groups, we plotted the risk score values of NRES and RES enrolled in the study on a scatter plot. The results, shown in Fig. 5E, indicate that the NRES population is homogeneous, with no clear discriminating differences in both groups as previously suggested in Fig. 5C.

**Fig. 6:**
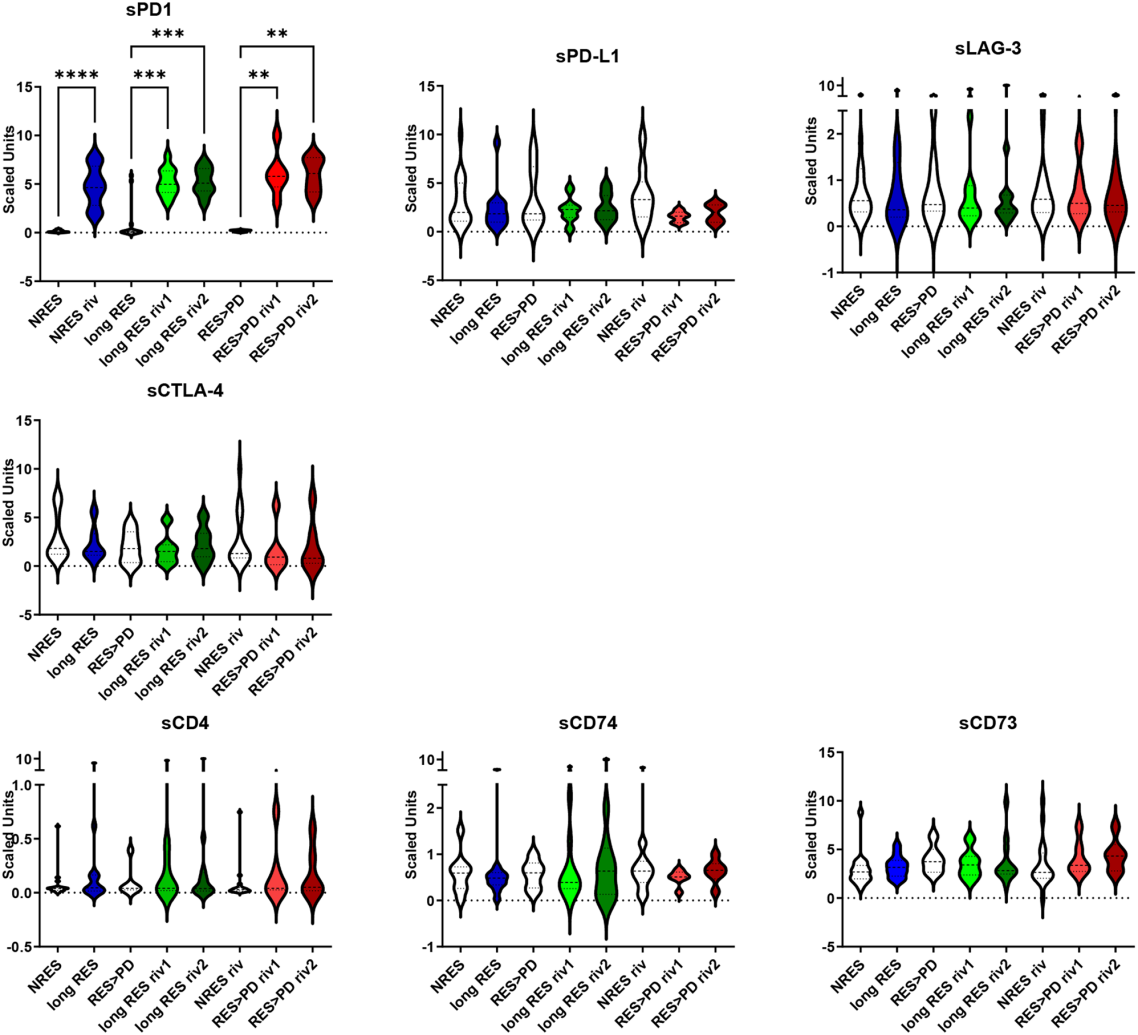
Circulating biomarkers clustered by response to therapy of MM patients. Violin plots with median of biomarker, expressed in scaled unit, at baseline, first and second revaluation from NRES (n = 18) long RES (n = 12) and RES>PD (n = 6) (Mann Whitney t test **p < 0.01, *** p < 0.001)

Furthermore, the analysis of each biomarker concentration at baseline among the 3 groups of patients showed that were not significant differences (Fig. S3). Conversely, considering the modulation of each biomarker in function of time, only for sPD1 a strong increase was found following immunotherapy, however with no differences in function of time among each of the three populations (Fig. 6).

## Discussion

Identifying minimally invasive biomarkers to help oncologists select optimal MM patients for specific cancer therapies remains a critical objective. This is particularly true if the therapy is immunotherapy, both due to the possible side effects that can worsen the patients’ quality of life and the high cost to the NHS. Currently, among the identified biomarkers, PD-L1 tumor score and tumor mutational burden (TMB) are the most established for clinical use, although they have some limitations. In the past we have already carried out a study to characterize the various populations of extracellular vesicles that can be used as predictors of response to anti-PD1 in metastatic melanoma [2, 3]. In this study, we focused on exploring the potential predictive role of certain soluble plasma factors, which could be directly measured without requiring preparatory steps for the plasma samples.

The four circulating immune checkpoints, sPD1, sPD-L1, sCTLA-4 and sLAG-3 and the three circulating immune-related proteins, sCD4, sCD73 and sCD74 were measured with Elisa method and analysed with bioinformatics approaches highlighting that (i) none of the biomarkers was statistically different between RES and NRES, even considering each response group (CR, PR, SD and PD); (ii) a higher number of significant correlations between biomarkers were observed in NRES compared to RES and (iii) by multivariate Cox regression, two independent factors sCD74 and sCTLA-4 were found as predictive for PFS and sCD74, sCTLA-4 and sPD-L1 for OS. This statistical approach allowed us to develop a risk model for disease progression. Following a rigorous cross-validation phase, the model was successfully applied to two carefully selected populations of MM patients.

The first analysis effectively distinguished the risk of progression in treatment-naive individuals, while, in patients who had undergone prior therapy, the model’s performance was less robust, even if a statistical trend was reached, likely due to the small sample size and the variability in pre-immunotherapy treatments, such as chemotherapy or targeted therapies (anti-BRAF/anti-MEK), sometimes in combination with anti-CTLA-4.

The latter is a small MM patient cohort whose clinical history was available and which included NRES, long RES (patients with a long positive response to therapy) and RES > PD (patients in whom, after an initial response the melanoma had progressed). Even if the population used was small and it will certainly be necessary to confirm the results in a larger cohort, the trend obtained demonstrated that higher values of the risk score were correlated with a lower PFS and that patients who progressed after an early response positive to the therapy, felt within the same range of risk score values of the NRES. Therefore, if the data and the hypothesis will be validated in a larger cohort of MM patients, the physicians could have a tool that would lead to identifying patients who would show a long-lasting response over time, because the risk model has the potential to categorize patients RES > PD as non-responsive to immunotherapy. The analysis of how each biomarker level changes in function of time demonstrated that, following anti-PD1 administration, no statistically significant changes were observed compared to pre-treatment samples, except for sPD1. The sPD1 concentration increased significantly post-immunotherapy; however, no differences were found among patient groups (NRES, long RES, or RES > PD). This indicates that sPD1 levels cannot be used for therapy monitoring. This result further supports our previous evidence showing that the tumour-derived extracellular vesicles positive for PD1 are monitoring biomarker of anti-PD1 response [3].

In literature, the topic of the study has been little investigated in melanoma. Other authors have demonstrated that sPD-L1 levels are significantly higher in melanoma patients compared to healthy individuals, especially in those with progressive disease [16] unlike our results, which showed no statistically significant differences between responders (RES) and non-responders (NRES). Zhou demonstrated that sPD-L1 levels did not appear to correlate with clinical response in the initial months; however, differences became evident after at least five months, particularly in patients with partial responses to anti-PD1 therapy [16]. These findings align with our data, which showed no significant variations in sPD-L1 levels within the first five months in NRES (median: 4 months) and during the first reassessment of long-term responders (RES). An increasing trend, though not statistically significant, was observed in sPD-L1 levels during the second reassessment after five months in long-term responders (RES) and in those who shifted from response to progression (RES > PD) (median reassessment times: 4–18 and 12–30 months, respectively).

The elevated levels of sPD-L1 and sPD1 in metastatic melanoma as respect to healthy have also been confirmed by Ugurel [17], who demonstrated that both immune checkpoints were present in the serum at higher concentrations before immunotherapy in non-responders. Ugurel reported that combined baseline serum sPD1 and sPD-L1 were considered predictors of anti-PD1 response [17], while in our analysis, sPD1 levels were not differentially expressed between NRES and RES. However, we employed a different approach, developing a risk score model and using independent factors from multivariate analysis to identify the score value for PFS, which allowed categorizing patients into RES and NRES groups.

Our choice of these biomarkers is further supported by the fact that these immune checkpoints have also been studied in cancers other than melanoma.

sPD1 and sPD-L1 have also been characterized as prognostic and/or predictive factors for immunotherapy response in non-small-cell lung cancer (NSCLC), pancreatic ductal adenocarcinoma (PDAC), and renal cell carcinoma (RCC) [18–20]. In PDAC, the prognostic role of sPD1 and sPD-L1 has been demonstrated, consistent with findings in other diseases [18]. In NSCLC, a similar trend to metastatic melanoma was observed, with higher levels in non-responders compared to responders, and no statistically significant modulation during therapy [21]. Later, Hayashi demonstrated that both circulating immune checkpoints, along with sCTLA-4, increased in non-responders compared to responders, especially in patients with tPD-L1 > 50% [19]. Additionally, the ability of sPD1, sPD-L1, and sLAG-3 to predict PFS and OS was studied in RCC, with only sLAG-3 emerging as a predictive factor [20], differing significantly from our results and thus suggesting a tumor-specific predictive role.

The literature data on other circulating immune checkpoints as predictive factors is scarce. sCTLA-4 has been shown to predict response to Ipilimumab in melanoma, with higher circulating levels in responders compared to those with progressive disease [22]. These findings, despite a different immunotherapeutic approach, are consistent with our results in melanoma responding to anti-PD1 therapy. Supporting the prognostic and predictive role of this circulating immune checkpoint are data from Liu and Teng [23, 24]. sCTLA-4 was evaluated as a prognostic factor in lung and esophageal cancers, demonstrating that increased post-therapy levels (chemotherapy or radiochemotherapy) correlated with longer OS and PFS, suggesting that this soluble factor may block the classic immunosuppressive activity of CTLA-4 [23]. Conversely, an opposite trend was observed in HCC patients receiving radiofrequency ablation [24]. Regarding sLAG-3 as a coinhibitory immune modulator in patients before and during immune checkpoint blockade, Gorgulho demonstrated that sLAG-3 was more highly expressed in patients than in healthy individuals, with elevated levels correlating with worse OS and PFS [25]. These findings contrast with the lack of significance in our study, potentially explained by a fundamental difference: our cohort included 110 melanoma patients, while Gorgulho’s study had only 11 melanoma patients out of a total of 84.

Data on sCD4, sCD73, and sCD74 as predictors of immunotherapy response are also absent or limited. According to Morello, sCD73 activity is higher in melanoma patients than in healthy individuals and greater in non-responders than responders, suggesting a potential role for sCD73 as a predictor of nivolumab response [26]. This finding was later validated in a larger patient cohort [27]. Our data contrast with these reports, possibly due to differing methodologies: their studies assessed sCD73 activity, while we measured sCD73 expression levels in plasma. Turiello et al. prioritized activity data over expression levels to achieve more accurate patient stratification [27]. This discrepancy raises an open question about whether sCD73 activity is directly correlated with its plasma levels in responders and non-responders, which we plan to explore in the future. For sCD74, it has been shown that sCD74 levels have a prognostic role in melanoma, correlating with tumor tissue CD74 expression. Patients with high sCD74 and low Macrophage Migration Inhibitory Factor (MIF) levels had better OS compared to those with low sCD74 and high MIF [15]. Our data further characterize sCD74 role in predicting response to anti-PD1 therapy in melanoma.

Notably, we found a higher number of significant direct correlations between the immune checkpoints and/or immune-related proteins in NRES compared to RES, that can be explained by the simultaneous expression/release of such molecules, which is consistent with the co-existence of multiple mechanisms of resistance to anti-PD1 therapy in NRES. NRES are often expected to exhibit a more complex network of immune checkpoint interactions, suggesting a tumor microenvironment that has adapted to evade immune surveillance by activating compensatory pathways to suppress the immune response. This may be rooted in an inherently dysregulated immune system, where checkpoints like sLAG-3, sPD-1, and sCTLA-4 play interdependent roles in sustaining an immunosuppressive environment [4, 19, 28]. Additionally, anti-PD1 resistance in these patients might involve alternative immune pathways becoming dominant, creating a coordinated resistance strategy [29, 30]. Finally, chronic inflammation or the presence of immunosuppressive regulatory cells like Tregs and MDSCs, which are often more pronounced in non-responders, may drive the simultaneous upregulation of multiple checkpoints, resulting in the observed stronger correlations [31–33]. The correlation between sPD1 and sLAG-3 in NRES and between sPD-L1 and sCTLA-4 in RES suggest an immunosuppressive environment in NRES compared to an immune-responsive one in RES. The inverse correlation between sPD-L1 and sCTLA-4 observed in RES is not representative of a hot tumor as suggested by Santos-Briz [34] because a “hot” tumor has a direct correlation between the two biomarkers reflecting their tumor expression. Thus, further investigations are warranted to determine the role of these soluble biomarkers in the TME. In NRES, sPD1 and sLAG-3 showed a direct correlation and it is known that PD1 and LAG-3 are often co-expressed on both exhausted T cells and tumor cells within the tumor microenvironment [19, 35]. Exhausted T cells are characterized by functional impairment and upregulation of multiple inhibitory receptors, including PD1 and LAG-3. The co-expression of these checkpoints indicates a state of deep T cell dysfunction and is associated with poor anti-tumor immunity [35]. Therefore, the observed direct relationship between the two circulating biomarkers, released by T lymphocytes and tumor cells, suggest that NRES are characterized by an immunosuppressive tumor microenvironment and a resistance mechanism. Additionally, the expression of other inhibitory receptors like LAG-3 could facilitate immune evasion [19]. Given the biological role of sPD-1 and sPD-L1 in restraining the activity and survival of T cells and Antigen-presenting Cells (APCs) [36], we can speculate that a direct correlation between such biomarkers is an additional evidence of the systemic immune tolerance in NRES. Further two significant correlations found in NRES are those between sCD73 and sCD74 and between sCD74 and sPD1. However, to the best of our knowledge, aside from evidence showing that both sCD73 and sCD74 are involved in cancer immune pathway, no data are currently available on a possible correlation between them in the context of the response to anti-PD1 therapy, which represents one the results of the present study.

## Conclusions

An unmet clinical need exists for easily accessible blood biomarkers that can be determined through simple analytical methods for predicting the efficacy of ICI therapy before treatment. In this study, we evaluated whether certain soluble factors could serve as potential predictive biomarkers for determining the effectiveness of immunotherapy in patients with MM. A combined biomarker score, reflecting the immune status of MM, proved to be more successful in predicting the response to anti-PD1 therapy than individual biomarker values, which tend to exhibit high variability.

## Electronic supplementary material

Below is the link to the electronic supplementary material.

Supplementary Material 1

## Data Availability

all data and material are available at the Laboratory of Experimental Pharmacology of IRCCS Istituto Tumori Giovanni Paolo II.

## Abbreviations

NHS: National Health System
EVs: extracellular vesicles
PoC: Point-of-Care
sPD1: Soluble Programmed Cell Death Protein 1
sPD-L1: Soluble Programmed Death-Ligand 1
sLAG-3: Soluble Lymphocyte-Activation Gene 3
sCTLA4: Soluble Cytotoxic T-Lymphocyte Antigen 4
sCD4: Soluble CD4
sCD73: Soluble CD73
sCD74: Soluble CD74
ELISA: Enzyme-Linked ImmunoSorbent Assay
PD1: Programmed Cell Death Protein 1
PD-L1: Programmed Death-Ligand 1)
LAG-3: Lymphocyte-Activation Gene 3
CTLA-4: Cytotoxic T-Lymphocyte Antigen 4
MHC: Major histocompatibility complex
MIF: Macrophage migration inhibitory factor
MM: Metastatic melanoma
PFS: Progression free-survival
RES: Responders
NRES: Non-responders
ICI: Immune-checkpoint inhibitor
CR: Complete response
PR: Partial response
SD: Stable disease
PD: Progression disease
OS: Overall survival
RS: Risk score
KM: Kaplan-Meier curves
TMB: Tumor mutational burden
NSCLC: Non-small-cell lung cancer
PDAC: Pancreatic ductal adenocarcinoma
RCC: Renal cell carcinoma
