# Supplementary material for: Developing a risk score using liquid biopsy biomarkers for selecting Immunotherapy responders and stratifying disease progression risk in metastatic melanoma patients
Source: J Exp Clin Cancer Res. 2025 Feb 5;44:40. doi: 10.1186/s13046-025-03306-w (PMC11796275; doi:10.1186/s13046-025-03306-w)
Supplement: Supplementary file 1 — Supplementary Material 1 [file 13046_2025_3306_MOESM1_ESM.docx]

**Supplementary figures**

**Fig. 1S Circulating biomarkers clustered by response to therapy in MM patients.** Scatter plot with median of the percentage of each biomarkers from MM patients with CR (n=13), PR (n=18) , SD (n=8) and PD (n=71).

**Fig. 2S Circulating biomarkers of MM patients clustered by evaluation of PFS and OS.** A. Kaplan–Meier survival curves according to each circulating biomarkers clustered by ROC cut-off as respect to PFS, and B. as respect to OS.

A

B

**Fig. 3S** **Circulating biomarkers of MM patients enrolled in the longitudinal study.** Scatter plot with median of the percentage of each circulating biomarkers at baseline from NRES (n=18) long RES (n=12) and RES>PD (n=6).

**Table 1S. Correlation analysis.** Statistically significant correlation results between each couple of circulating biomarkers of RES and NRES obtained using the nonparametric Spearman correlation test (*p < 0.05, **p < 0.01, *** p < 0.001).

| **Patients** | **Circulating biomarkers** | **Spearman r** | **95% confidence interval** | **p value** | **p value summary** |
| --- | --- | --- | --- | --- | --- |
| RES | sPD-L1 and sCTLA-4 | -0.5885 | -0.7770 to -0.3030 | 0.0003 | *** |
|  | sLAG-3 and sCD4 | 0.7094 | 0.4804 to 0.8479 | <0.0001 | *** |
|  | sCTLA-4 and sCD73 | 0.3966 | 0.05700 to 0.6539 | 0.0202 | * |
| NRES | sPD1 and sPD-L1 | 0.3409 | 0.1183 to 0.5309 | 0.026 | ** |
|  | sPD1 and sLAG-3 | 0.3760 | 0.1578 to 0.5591 | 0.0008 | *** |
|  | sPD1 and sCD4 | 0.2838 | 0.05549 to 0.4839 | 0.0130 | * |
|  | sPD1 and sCD74 | 0.3308 | 0.1071 to 0.5227 | 0.0035 | ** |
|  | sPD1 and sCD73 | 0.2562 | 0.2576 to 0.4607 | 0.0255 | * |
|  | sLAG-3 and sCD4 | 0.6629 | 0.5093 to 0.7756 | < 0.0001 | *** |
|  | sCD4 and sCD73 | 0.2416 | 0.01028 to 0.4484 | 0.0355 | * |
|  | sCD74 and sCTLA-4 | 0.2578 | 0.2753 to 0.4621 | 0.0245 | * |
|  | sCD74 and sCD73 | 0.3239 | 0.09945 to 0.5170 | 0.0043 | ** |
